# How long to rest in unpredictably changing habitats?

**DOI:** 10.1371/journal.pone.0175927

**Published:** 2017-04-18

**Authors:** Mirosław Slusarczyk, Jacek Starzyński, Piotr Bernatowicz

**Affiliations:** 1 University of Warsaw, Faculty of Biology, Department of Hydrobiology at Biological and Chemical Research Centre, Warsaw, Poland; 2 Institute of Theory of Electrical Engineering, Measurement and Information Systems, Warsaw University of Technology, Warsaw, Poland; 3 University of Warsaw, Faculty of Biology, Department of Paleobiology and Evolution at Biological and Chemical Research Centre, Warsaw, Poland; 4 University of Warsaw, Faculty of Biology, Department of Animal Physiology, Warsaw, Poland; University of Thessaly School of Agricultural Sciences, GREECE

## Abstract

In the present study, we investigated the optimum length of prolonged dormancy (developmental arrest extending over favourable periods) of organisms under uncertain environmental conditions. We used an artificial life model to simulate the evolution of suspended development in the ontogenesis of organisms inhabiting unpredictably changing habitats. A virtual population of semelparous parthenogenetic individuals that varied in a duration of developmental arrest competed for limited resources. At a constant level of available resources, uninterrupted development was the superior life strategy. Once population fluctuations appeared (generated by the stochastic variability of available resources), temporal developmental arrest became more advantageous than continuous development. We did not observe the selection of the optimum length of dormancy, but rather the evolution of a diversified period of developmental arrest. The fittest organisms employed bet-hedging strategy and produced diversified dormant forms postponing development for a different number of generations (from 0 to several generations, in decreasing or equal proportions). The maximum length of suspended development increased asymptotically with increasing environmental variability and was inversely related to the mortality of dormant forms. The prolonged dormancy may appear beneficial not only in erratic habitats but also in seasonal ones that are exposed to long-term variability of environmental conditions during the growing seasons. In light of our simulations the phenomenon of very long diapause (VLD), lasting tens to thousands of generations, which is occasionally observed in ontogenesis of some living creatures, may not be explained by the benefits of bet-hedging revival strategies. We propose an alternative reasoning for the expression of VLD.

## Introduction

All environments are subject to change either for abiotic or biotic reasons. Environmental fluctuations may differ between sites in their origin, amplitude and degree of predictability. Three major mechanisms are used by living creatures to cope with environmental fluctuations: a) physiological flexibility [1], b) spatial [2, 3], or c) temporal avoidance of environmental change [4, 5, 6, 7]. All living creatures possess physiological mechanisms which let them maintain internal integrity despite fluctuations of their habitat. Homeostatic capabilities differ between species and ontogenetic stages of a given species; however, they are always limited. When the intensity of environmental change exceeds the tolerance capacities of living creatures, they may survive via spatial or temporal avoidance of unfavourable conditions. Although most animals actively seek favourable sites, few possess effective movement capabilities which let them inhabit favourable habitats throughout their whole lives by temporal switching between fluctuating environments (e.g. some migratory birds). However, most organisms (regardless of being mobile or not) apply an alternative remedy–programmed developmental arrest, called diapause in animals and physiological dormancy in plants, which lets them survive extreme environmental fluctuations in their native habitats not tolerated by their active forms. In the present study, we will use the terms diapause, dormancy and suspended development interchangeably, to refer to the periodic mechanisms of developmental arrest and extend them to all living creatures regardless of their taxonomic classification. Diapausing individuals can commonly tolerate a wider range of environmental changes than active forms, due to a periodic suspension of one or a few living functions: development (the only mandatory option), growth, food processing, movement, metabolic activity, etc. [8]. Compared to active forms, diapausing forms, due to limited functionality and lower demands, may utilise less effective metabolic pathways, that, however, offer internal integrity at a broader range of environmental conditions. Diapause is often associated with metabolic retardation, though this is not a mandatory feature (*vide* reproductive diapause [9]).

Diapause is regarded as a costly adaptation. It has been suggested that needless suspension of development and reproduction may have exposed genetic lines of organisms to competitive exclusion by individuals that complete diapause in a shorter suitable period or develop directly [10, 11, 12]. Diapause may affect the vigour of active forms prior to or after developmental arrest: by lowering fecundity [13, 14] or shortening longevity (e.g. by exposing organisms that form dormant stages to a higher risk of being preyed upon [15]). Longer diapause periods would require higher metabolic suppression, more abundant reserves, or both. Finally, although dormant forms are typically more resistant to extreme environmental conditions than active forms, mortality risk may accumulate over the dormancy period. All these factors may explain why diapause is utilised mostly for short periods, in predictably changing (e.g. seasonal) habitats [4, 8]. Diapause typically begins just in advance of expected environmental deterioration [11, 16] and ends shortly after conditions improve again [8]. However, few habitats change in a predictable manner and the degree of unpredictability of environmental fluctuations may affect the timing of the developmental arrest. While most dormant forms are only able to suspend development for short periods (not longer than a single unfavourable season), some organisms may thrive in diapause for years, decades, or even centuries (see [17] for a comparison of diapause duration in crustaceans). Evidenced records claim a diapause duration of 700 years in the crustacean *Daphnia* [18], 1,300 years in the water plant *Nelumbo nucifera* (Nelumbonaceae) [19], 2,000 years in the terrestrial plant *Phoenix dactylifera* (Arecaceae) [20] and millions of years in the cases of some bacteria [21, 22]. It is unclear if such long periods of dormancy have any adaptive value or rather that they are some kind of artefacts.

We can imagine at least two different adaptive reasons for the evolution of prolonged diapause, sometimes called Extra Long Diapause (ELD): diapause that extends over favourable seasons. Both are related to the temporal unpredictability of environmental conditions but acting on different life stages of organisms: 1) dormant, or 2) active organisms.

The first reason mostly concerns motionless diapausing stages that are used for spatial dispersal and may be intentionally or unintentionally displaced into unfriendly microhabitats for an indefinite period of time. Plant seeds or resting forms of animals left in/on soil or sediments may be covered for long periods by impermeable deposits, before some external force brings them back eventually to the surface where their further development is feasible. It seems that long-term diapause of immobile dormant forms facilitates persistence in spatially and temporally varying habitats until an occasion arises for development.

The second reason (which we are focussing on in the present study) differs fundamentally from the first and concerns the challenges of active forms with the unpredictability of living conditions in their native habitats. Irregular changes of living conditions may occur not only in ephemeral habitats but also in a wide array of more predictable sites. Many habitats which change predictably on a daily or seasonal time scale are less predictable over a longer (e.g. multiannual) time perspective. In turn, we may observe large population fluctuations of organisms between years for various abiotic or biotic reasons [23, 24, 25], which are impossible to anticipate in advance. If all dormant forms of organisms present in a habitat resumed development in the first favourable season, a single growing season with low survival or a failed reproduction (in the case of annuals) of active forms would decimate or even exterminate the entire population. To reduce this risk, various precautions may be employed by organisms, and one could be a bet-hedging revival strategy of dormant forms [4, 6, 26]: this is where only a portion of diapausing stages formed by cautious species resume development at the first occurrence of favourable conditions while the rest wait until the next or even later time windows suitable for development. How long should organisms postpone development in unpredictably changing habitats? This question has rarely been considered and is the issue we targeted in the present study.

The optimum period of diapause termination was first theoretically investigated by Cohen [4], who concluded that in predictably changing habitats diapause should cease shortly after environmental conditions improve again. Diapause may be prolonged until the second of further favourable seasons to reduce the consequences of failed reproduction in habitats changing in less predictable ways. The less predictable the seasonal fluctuations are, the lower the fraction of diapausing forms that should terminate diapause in the first favourable season [4]. Cohen’s conclusions were supported by further theoretical [5, 27, 6] and empirical [26, 28] studies. While Cohen mentioned [4] that developmental arrest may last longer than a single environmental cycle, longer periods of diapause have so far rarely been considered. One of a few was Menu with co-authors, who considered the effect of longer periods of diapause in theoretical [29, 30] and empirical [31] studies on life cycles of a terrestrial insect–the chestnut weevil (*Curculio elephas*). They concluded however, that in the studied case 1–2 environmental cycles in diapause were more beneficial than 3 or more. In the present study, we aimed to analyse whether longer periods of diapause could offer any benefits to living creatures under any circumstances: we used computer-assisted simulations to test this phenomenon.

## Methods

### Model description

We used individual based model, sometimes called an artificial life model [32], to simulate the evolution of suspended development in the ontogenesis of parthenogenetic and semelparous individuals residing in habitats with available resources fluctuating unpredictably each generation. In our model diapause appeared at early ontogenetic stage—in form of resting eggs. Our prediction should be valid in other circumstances as well, e.g. in sexually reproducing organisms and for diapause occurring at later stages of ontogenetic development. Virtual individuals consumed resources and produced eggs that developed directly or entered diapause for various periods of time. Diapausing and active individuals could survive until the next generation, with a defined probability, different for each type of individual. The survivorship of dormant eggs and its effect on the ultimate length of diapause was one of the investigated parameters. In most simulations we assumed that mortality of dormant eggs was constant per unit of time (5% per generation); thus, survivorship of dormant forms decreased exponentially with time spent in diapause. The probability of survival and reproduction of active individuals decreased as the population density approached the environmental capacity according to the formula p = 1-N/K, where p—reproduction probability, *N*—number of individuals in the population, *K*—environmental capacity. Environmental capacity was changed randomly during each generation within a tested range of possible values that were the subject of investigation. The range of possible population numerical changes was expressed in terms of standard deviations of the environmental capacity. The dormant eggs accumulated in a germ bank with time. The time of egg activation was an individual feature and was randomly assigned from the inherited distribution encoded for each individual.

The offspring activation period was the most important parameter in our model. It had a range of assigned values from 0 to 49 generations (where 0 means activation in the next generation, while values from 1 to 49 stood for longer periods of diapause, in terms of generations). The actual value was randomly drawn from the distribution, which featured each individual. The dormancy distribution could have been set in any possible way. It was possible to activate 100% of offspring in one particular season or distribute them evenly in the following seasons (e.g. 2% in each of the 50 following seasons). Other distributions between these two extremes were also possible.

Each offspring inherited its activation probability distribution from its parent. The inherited distribution was exposed to a mutation process with defined probability. We assumed a mutation probability of 0.00001 per tested feature in each generation, which remains within the range suggested by Willensdorfer *et al*. [33]; however, mutation probability had only a marginal effect on the key results of the study (S1 Fig). The probability distribution for offspring activation was limited by the parameter d_max_ (maximum time spent in diapause). This parameter had a range of assigned values from 0 to 49 generations. Moreover, as in the case of the activation distribution, the maximum time spent in diapause could have been affected by mutation during simulation (with the same probability and step of change). At the beginning of each simulation we assumed a flat population structure of individuals forming progeny that remained in diapause from 0 to max 49 generations in equal proportion. Thanks to mutation (of d_max_ as well as the egg activation distribution) and competition between the tested strategies for limited resources it was possible to observe virtual evolution of dormancy strategies under the tested conditions.

**Fig 1:**
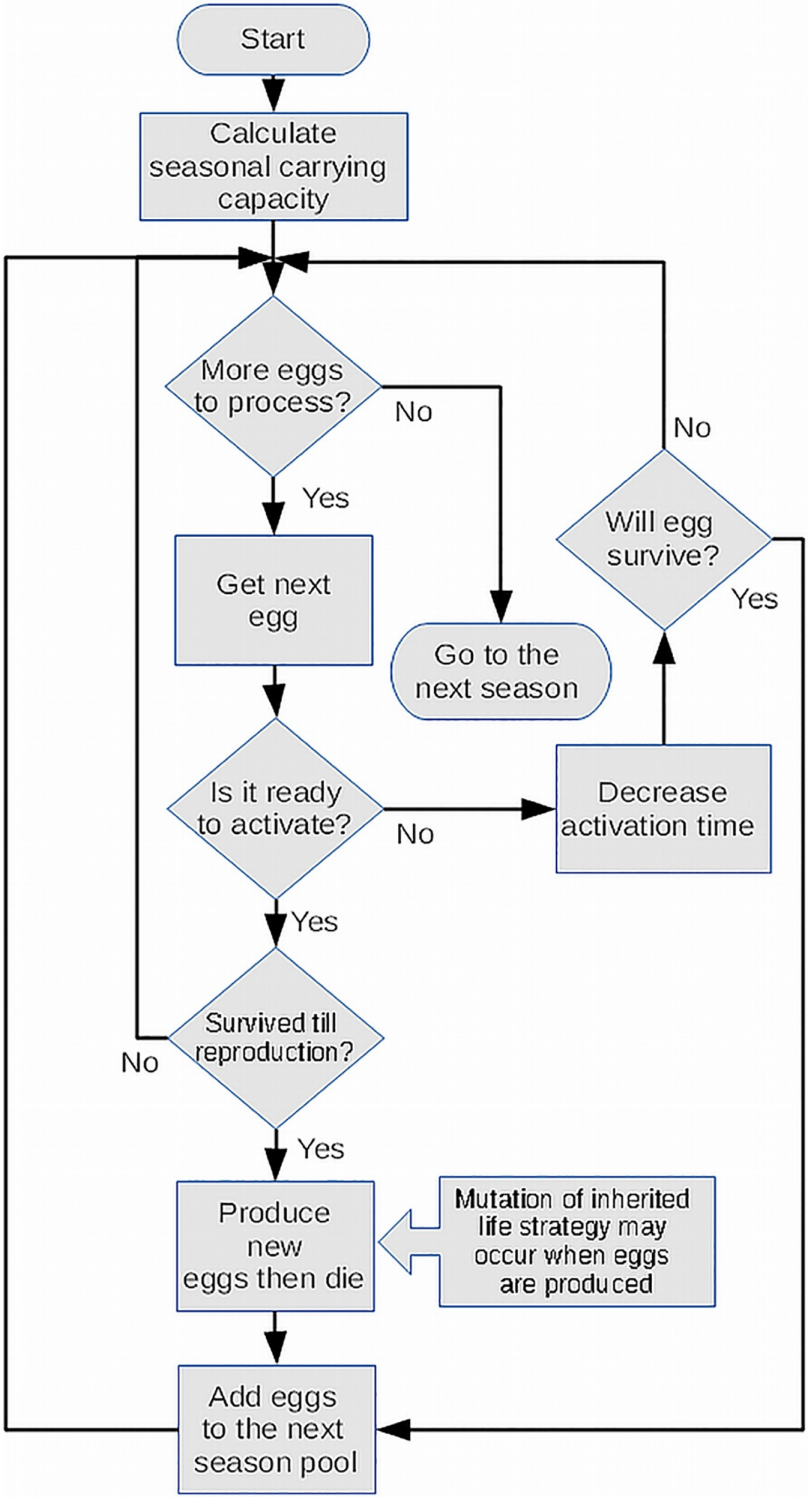
The scheme of a one generation cycle of the virtual population of our simulation.

A simulation scheme of one generation loop of the program is shown in the Fig 1.

At the beginning of each (non-overlapping) generation the program calculated the carrying capacity of the habitat–K. The value of K was determined randomly from a tested range as stated above. The main part of the simulation was an "individual" loop. Each egg from the egg bank was checked if it was ready for activation in a given generation according to its inherited pattern. If it was not ready it was moved to the next generation’s egg pool, and its inherited activation time was reduced by one generation. If the egg was ready for activation, it was determined whether it survived until reproduction according to the formula p = 1-N/K. If the activated egg did not survive until reproduction, it was deleted from the simulation. Otherwise the activated egg developed, produced a fixed number of eggs (6 in most simulations) and then died. During egg production, a random mutation of d_max_ and inherited activation pattern could have occurred for each egg at the defined rate mentioned above. The newly formed eggs were moved to the next generation’s egg pool. The "individual" loop was repeated for each egg present in the egg bank pool. The eggs in the egg pool were exposed to external mortality at the rate mentioned above. The description and values of experimental parameters can be found in the S1 Table.

Each generation faced a different food quantity assigned randomly from the same distribution. Environmental capacity could not be negative: if negative values randomly appeared, we changed them into 0.

**Fig 2:**
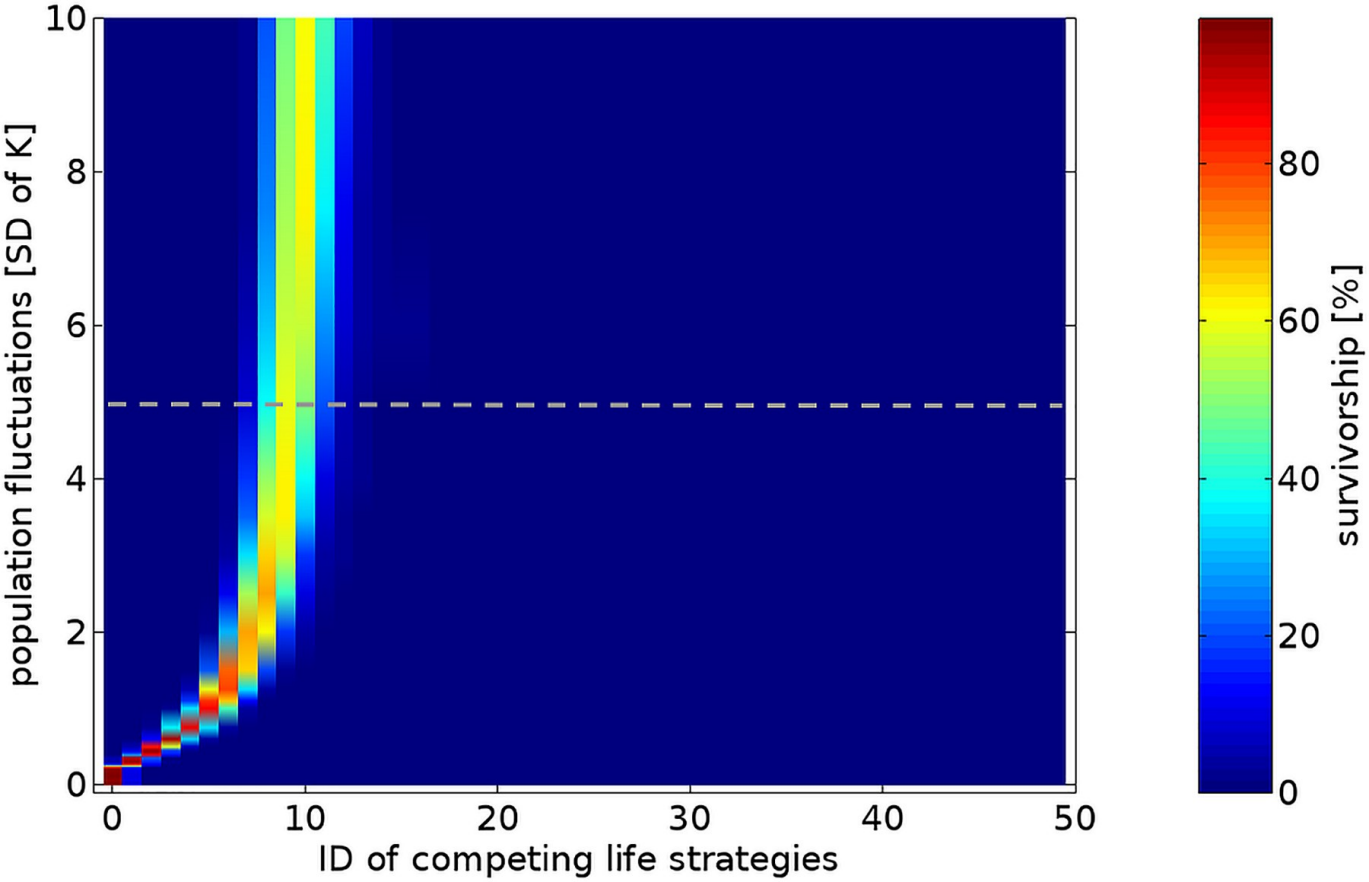
Mean survivorship of various life strategies competing for limited resources for 5,000 generations at different range of environmental variability when mortality of dormant forms assumed as 5% per generation. The strategies differ in maximum lifespan of developmental arrest of the diapausing forms. Population fluctuations are presented as relative values of standard deviations of the carrying capacity. The dotted line indicate cross-section presented on the next graph.

**Fig 3:**
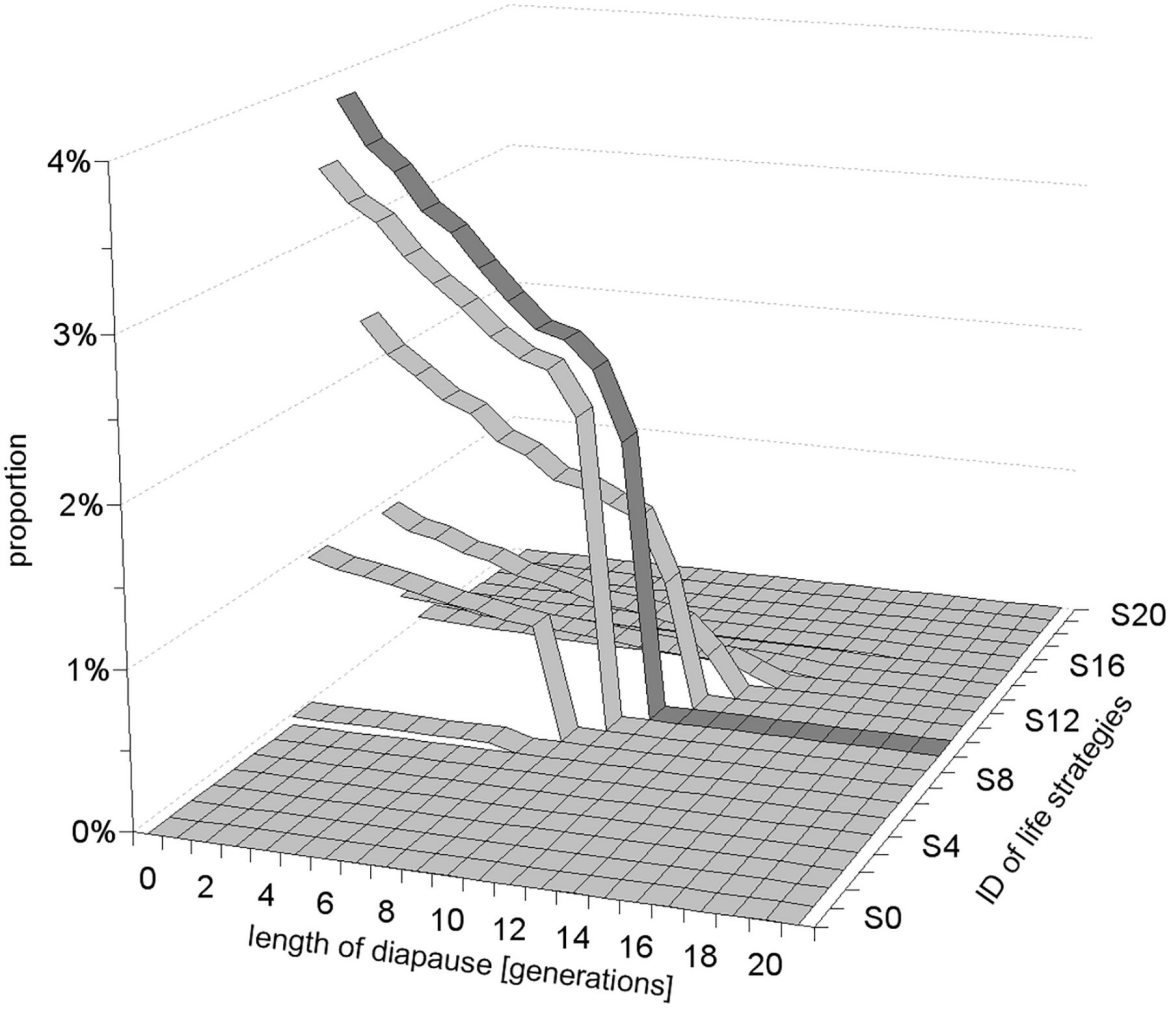
The example of a final structure of dormant stages formed at the end of simulation by most successful life strategies at high population fluctuation SD = 5K and 5% mortality of dormant forms per generation, indicated by the dotted line on the Fig 2. Note that the d_max_ value—the longest diapause in the tested conditions is 17 generations, but it does not offer the highest advantage. The most successful strategy appeared here the strategy number 10 (indicated by dark grey colour).

**Fig 4:**
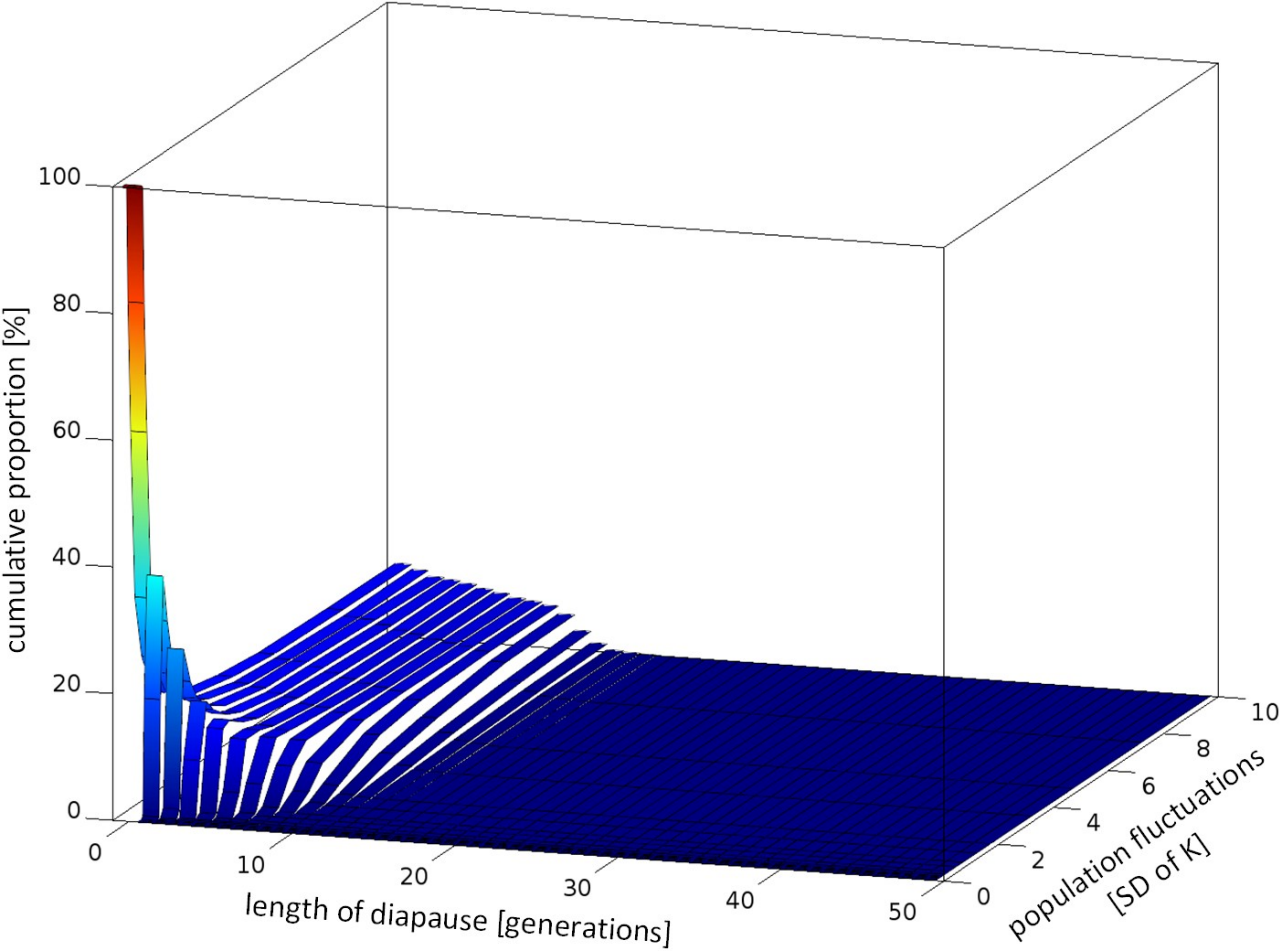
Cumulative (added up to 100%) proportion of dormant stages, inactive for various number of generations (0–49) formed by all surviving life strategies at the end of the competition experiments at various ranges of population fluctuations and 5% mortality of dormant forms per generation.

**Fig 5:**
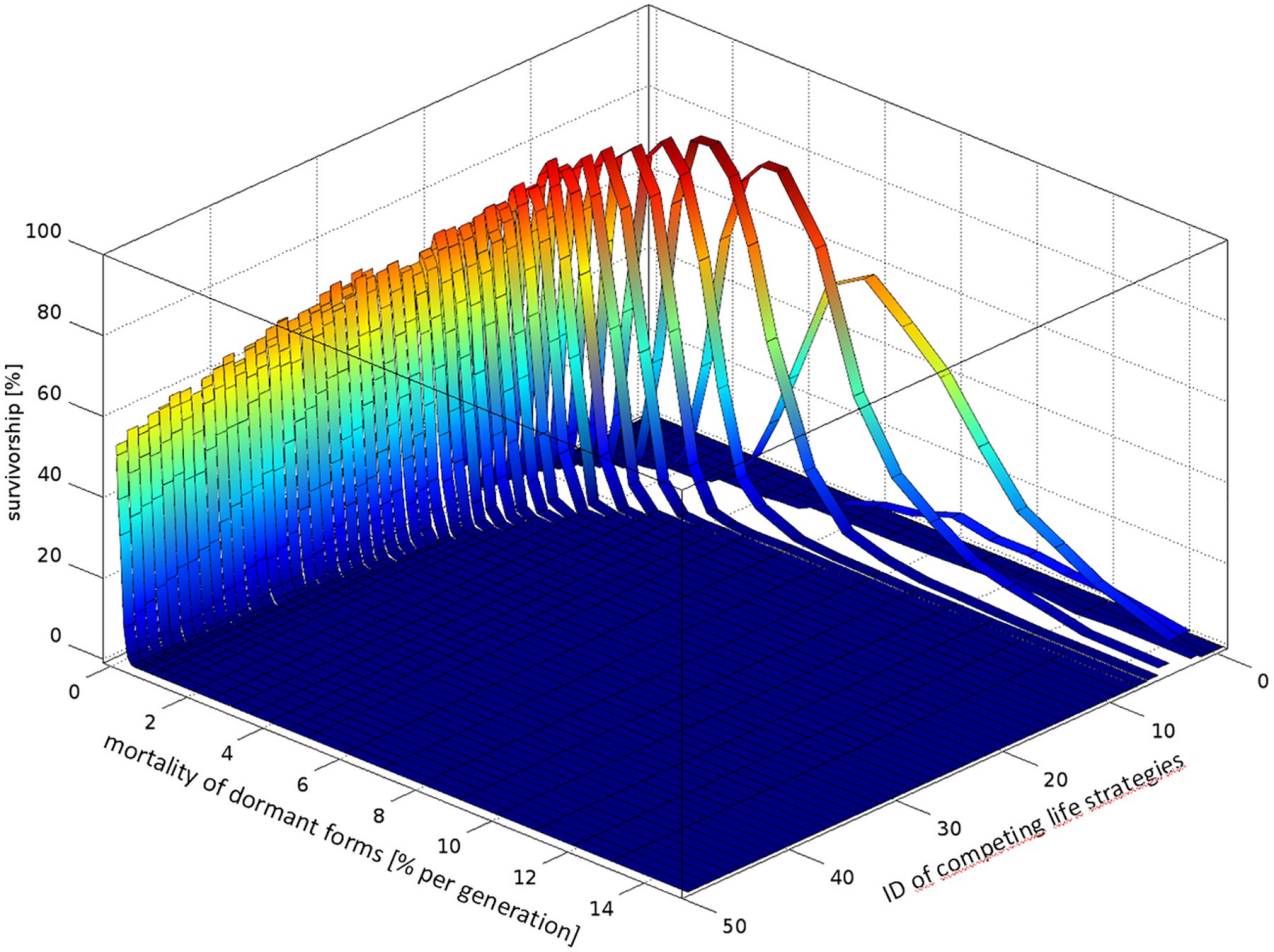
Effect of mortality of dormant forms on mean survivorship of life strategies competing for randomly varying resources for 5,000 generations at considerable population fluctuations (when SD = K).

The outcome of our simulation was the following: the quantity of offspring and probability distribution of their activation in each season. The derivative of these results was data about the survival of particular strategies. These data were used to draw Figs 2–5.

The computer simulation was written in C language, using a simple data structure and self-written dynamic memory management to obtain the highest possible speed. This approach allowed us to carry out all experiments on a common PC in reasonable time (dozens of minutes to dozens of hours per simulation, depending on the input data). The program can be downloaded and tested [34].

We carried out two independent experiments. We tested the effect of: (A) environmental variability and (B) mortality of dormant forms on the evolution of life strategies. Each simulation lasted 5,000 generations and was repeated 1,000 times. The probability distributions for the occurrence of particular environment capacity values are shown on the S2 Fig.

We performed most simulations with relatively low environmental capacity values (K = 500), which allowed us to reduce computation time significantly. We found that K values had a marginal effect (except very low values of K < 25) on the result of optimal dormancy strategy evolution, once the possible range of population fluctuations were expressed in relative values of K–i.e. as SD of K. See S3 Fig for comparison.

## Results

In invariant habitats individuals producing directly developing—strategy #0—always won the competition with other strategies producing ELD forms (i.e. strategies #1 or higher) (Fig 2).

At low environmental fluctuations, the strategies producing short term dormant stages coexisted with strategy #0. As environmental variability increased (SD ≥ 0.5K) strategy #0 was outcompeted by strategies producing short term or long term dormant forms (Fig 2). With increasing fluctuations of population quantity, the length of diapause of the successful life strategies (surviving in our simulations for the investigated period of 5,000 generations) increased asymptotically towards a maximum value (D_max_) determined by the mortality of dormant forms (Fig 2). At lower mortality of dormant forms somewhat longer diapause periods were beneficial (for comparison see S4 Fig). Interestingly, the successful strategies, including the most predominant ones in given environmental conditions, did not produce just a single type of dormant stage (i.e. optimum length of diapause) but formed diversified types of offspring—which remained in diapause for various numbers of generations from 0 to some max value in declining proportions (Fig 3) at high or moderate mortalities of dormant forms, or at almost equal proportions at lower mortalities (S5 Fig).

All successful strategies produced dormant forms that remained in diapause for various numbers of generations, from 0 to the local d_max_ value (the longest adaptive period of diapause in the given conditions) in declining proportions (Fig 4). The slope of this decline was strongly affected by the mortality of dormant forms (S6 Fig).

The maximum period of diapause used by the most successful strategy was slightly lower than the local d_max_ value reported under the given conditions (Fig 3). The local d_max_ period increased with increasing values of population variability (Fig 2) on the one hand, and the survivorship of dormant forms on the other (Fig 5).

At intermediate mortality of dormant forms (5% per generation) and extreme variability of population fluctuations (SD = 10K) the d_max_ value reached 18 generations merely. On the other hand, at extremely low mortality of dormant forms (0.1% per generation) but intermediate value of population fluctuations (SD = 1K) the d_max_ reached the utmost limit tested in our study: 49 generations. At low mortality of dormant forms, unlike at higher values, it was difficult to determine the most successful diapause strategy as the strategies producing very long lasting and variable dormant forms (VLD) appeared as successful as strategies forming somewhat shorter and less variable dormant forms (Fig 5). Therefore, intraspecific competition for resources between coexisting strategies reduced survivorship of each other below the survivorship values reached by the predominant strategy when survivorship of dormant forms was lower. For these reasons, at considerable population fluctuations (SD = 1K) the highest survivorship of the most successful life strategy was reported not at the lowest, but at some intermediate mortality of dormant forms (1–5% per generation, Fig 5). Interestingly, ELD appeared as a more successful strategy (strategies #1 or higher) than direct development (the strategy #0) not only at high environmental fluctuations with a high risk of failed reproduction of active forms in subsequent generations, but also in less variable habitats, when carrying capacity (K) was always positive, e.g. when SD = 0.2K (Fig 2).

## Discussion

The results of our simulations indicate that neither short nor long-term diapause is advantageous in constantly favourable conditions. Organisms which produced directly developing offspring eventually outcompeted individuals that employed any kind of dormancy (Fig 2). This is not surprising and has been claimed before [4, 6]. Apparently, diapause was not beneficial at constant conditions while it generated substantial mortality costs. In invariant habitat, when we reduced mortality of dormant forms to zero, direct development remained the best option; however, organisms postponing development for one or more generations were not excluded but rather coexisted with the former at a somewhat lower proportion (S7 Fig). This indicates that the main component of diapause cost is the mortality of dormant forms, and not a reduction of organism proliferation rate, as is frequently claimed. Since null mortality of diapausing forms is a theoretical rather than real scenario, in our study we used positive values for this parameter. In most simulations we applied a constant mortality rate of dormant forms per generation (5%); this seemed like the most reasonable pattern of their mortality, yet this is surely not the only pattern which occurs in nature. ELD (extra long diapause) became advantageous once unpredictable fluctuations in carrying capacity appeared and mortality of dormant forms was not substantial (Figs 2 and 5). During low population fluctuations, individuals producing only directly developing offsprings coexisted with individuals producing both short term dormant forms and directly developing offsprings. However, when fluctuations increased, the former were outcompeted by the latter (Fig 2). Apparently, at some threshold level of numerical fluctuations the long term benefits of diapause outweighed its short term costs. The higher the fluctuations in carrying capacity, the larger the benefits of ELD and longer periods of diapause were adaptive (Fig 2). What kind of benefits could suspended development, especially long term diapause, offer in fluctuating habitats? The consequences of occasional poor growing seasons (i.e. low reproduction or high mortality of active forms that cause their numerical decline) may be compensated for by the resurrection of diapausing survivors formed in preceding generations. ELD flattens population peaks, reduces competition for limited resources and, most importantly, fills the numerical troughs with the time travellers after unfavourable periods. Resurrection of diapausing stages may reduce the fatal consequences of decimation or extermination of the active forms and revive the genetic line. Long term diapause may be more effective in filling numerical gaps than short term developmental arrest, due to the higher number of time travellers originating from a more abundant population in the distant past compared to a recent population from periods of population decline, i.e. when the time travellers are most significant. In a more formal way, suspended development and especially long term diapause may offer benefits due to a reduction in numerical fluctuations of genetic lines over generations, which maximises the long term geometric mean of their fitness at the cost of the arithmetic mean of this parameter in unstable habitats [6, 35].

Our simulations allowed organisms to form variable types of progeny that remained in diapause from 0 to 49 generations. Strategy #0 in our simulations may, according to interpretation, refer either to directly developing organisms in invariable habitats or to individuals forming seasonal dormant forms during inhospitable periods in seasonally fluctuating habitats, i.e. individuals which are active whenever possible. Strategies # 1 to 49 form dormant stages that remain in diapause for one or more generations respectively. The life strategies in our simulations evolved from an initial flat structure (with the random proportion of different types of progeny) towards the ESS (Evolutionary Stable Strategy) due to selection and mutation. In turn, in randomly changing habitats we did not observe the evolution of a single optimum period of diapause but rather the formation of a set of offspring postponing development for various numbers of generations from 0 to some maximum value (d_max_) in a declining proportion; i.e. most offsprings developed directly, fewer resumed development after one generation, while still fewer after two, three or more generations (Fig 3). At low mortalities of dormant forms, the most successful strategies formed almost equal proportion of descendants remaining in diapause for a various number of generations (S5 Fig). These patterns can be classified as a kind of diversified bet-hedging revival strategies, following Phillippi and Seger [36].

In our model we assumed arbitrarily that each individual, if it was lucky enough to survive until maturation, formed 6 offspring. Each of these 6 offspring could remain in diapause for the same or different periods of time. The actual structure of time spent in diapause by descendants of each life strategy was subject to evolutionary change. In our simulation, the application of relatively low fecundity narrowed the number of dormancy categories revealed by descendants of a single parent. Yet bunches of clonal parents sharing a common life strategy could form a full array of descendants with various possible combinations of diapause periods from 0 to 49 generations. It is important that neither lower nor higher fecundity changed the main conclusions of our simulations except at very low values (≤2 offspring per clutch) (see S8 Fig for comparison).

The maximum period of diapause (d_max_) as well as the number of diapause categories of the dormant forms of the ESS were determined by two factors in our simulations: mortality of dormant forms on the one hand, and environmental variability on the other. At considerable levels of population fluctuations (when standard deviation of the carrying capacity matched the carrying capacity, i.e. when SD = K), diapause appeared more effective than direct development unless the mortality of dormant forms became substantial (>40% per generation) (Fig 5). As environmental fluctuations increased at a given mortality pattern of dormant forms, the maximum length of diapause (d_max_) and the number of categories of dormant forms employed by the most successful life strategies increased asymptotically to some maximum value (Fig 2). According to our simulations the maximum length of diapause (d_max_) of the ESS seems to be more affected by the mortality of dormant forms than by environmental variability. At moderate levels of mortality of dormant forms (5% per generation) and high fluctuations of population density (SD = 10*K), the d_max_ value reached 18 generations. Further increases in population size variability did not increase the d_max_ value significantly.However, when we reduced the mortality of dormant forms close towards zero, d_max_ reached the maximum allowed value in our simulations (49 generations) at a moderate level of population fluctuations (when SD = 1*K; Fig 5). Interestingly, at a very low level of mortality of dormant forms (≈0), the longest diapause strategy tested (strategy #49) appeared as successful as other life strategies with a shorter maximum period of diapause and a lower number of diapause categories (e.g. strategies #20–48), indicating no advantage of very long diapause (VLD—diapause lasting dozens of generations) over somewhat shorter diapause periods in variable habitats, and thus no ESS could be suggested. This implies however, that the evolution of VLD is feasible at low mortality levels of dormant forms. Interestingly, equal competence and coexistence of long term diapause strategies (strategies #20–49) at low mortality of dormant forms reduced the survivorship of each other below values reached by the most successful strategy when a higher mortality of dormant forms was applied, which may seem counterintuitive (Fig 5).

In light of the above arguments the reported records of diapause lasting centuries or even millennia in organisms of relatively short generation times [18–22] were not likely selected as the benefits of a bet-hedging revival strategy in variable habitats. We should rather seek alternative explanations for the evolution of this phenomenon, e.g. the spatio-temporal challenges of dormant forms in surviving until first occasion for development. In our opinion, the VLD may be selected for by long unfavourable periods faced by dormant forms that accumulate in unfriendly habitats for an indefinite period of time. This typically happens to passively dispersing dormant forms. Indeed VLD lasting decades, centuries, or millennia seems to be a feature of organisms that form immobile dormant stages designed for spatial dispersal that are exposed to unpredictable spatio-temporal variability of environmental conditions. Organisms that migrate in the active stage and use ELD forms primarily/exclusively for temporal dispersal rather do not spend more than a few generations in diapause (e.g. insects, according to Hanski [37]).

Interestingly, our simulations indicated that ELD may be adaptive at relatively low levels of environmental variability. Short term ELD appeared to be beneficial over direct development at relatively low values of population fluctuations, when SD = 20% of K merely, i.e. when the carrying capacity always remained positive (higher than 0) (Fig 2). Apparently, mere fluctuations of population density and thus variability in reproductive success of individuals may favour short term ELD when no risk of extermination of the entire population exists. This indicates intraspecific competition for resources as a potential driver of ELD evolution.

The largest existing dataset on ELD concerns the seeds of vascular plants and diapausing forms of insects, because of their economic values. We must remember that most vascular plants are sessile organisms and use their dormant forms not only for temporal but also for spatial dispersal. These two needs may pose different selective pressures on seed longevity and germination pattern [38], while in our model we only tested one of the two. However, if we insist on using vascular plants for model verification, annual plants would be the better choice rather than perennials, since the latter, in their ontogenesis, possess more than one dormant stage that copes with temporal variability, which may obscure data interpretation. Annual plants from risky desert habitats are known for diversified bet-hedging germination of their seeds [39, 40, 26, 41, 42]. Long term data on germination of desert winter annuals indicated that the germination fraction decreased with increasing variability of environmental conditions [40, 41] on the one hand, and mortality of seeds on the other [42], that supports the prediction of our model. In the vast literature on seed dormancy we could hardly find information concerning the germination pattern of seed cohorts of annual plants in consecutive seasons to compare our predictions with real data. One of the few available studies indicated various germination patterns of seeds of annual plants in two consecutive years [26]: seeds of a few genera (*Haplopappus*, *Lepidium*, *Plantago*) germinated at a higher proportion in the first than in the second year of the study, while in contrast another genus (*Microseris*) germinated at a lower proportion in the first year, whereas another (*Erigonum*) germinated at a comparable fraction in the two consecutive years. We do not know of any study that investigated the proportion of germinating seeds over more than two consecutive generations. For testing the prediction of our simulation, insects might be a better choice than plants as most of them disperse in their active stage and use their dormant forms for temporal dispersal exclusively. Moreover, most of them, being short living creatures, utilise a single diapausing stage in their ontogenesis. Indeed, some insects reveal a bet-hedging pattern of reactivation of diapausing forms in subsequent years. The majority of dormant stages of the gall midge *Contarinia sorgihicola* (Diptera: Cecidomyiidae) reactivated during the first favourable season (74%), much fewer reactivated in the second season (23%) while very few reactivated the third (3%) [43]. A similar pattern was reported for some other insects too [44, 31]. This pattern (a relatively short maximum period of diapause and sharp decline of the proportion of dormant forms over time) was expected in our model in relatively invariable habitats, when SD = 0.3 K. In more variable habitats our simulations expected a longer maximum period of diapause and a more gradual decrease in the proportion of dormant forms over time (Figs 3 and 4). The maximum period of ELD in motile insects that use dormant forms for temporal dispersal [37] is relatively short compared to plants or aquatic crustaceans, which both, unlike insects, use their dormant forms for both temporal and spatial dispersal, which may agree with the thesis stated above.

Another group of organisms that form a long term bank of resting stages are planktonic crustaceans and rotifers. Their small body size and short lifetime might be useful for empirical verification of our model. Unfortunately, both groups, as do plants, use their diapausing forms for spatio-temporal dispersal [45] which may obscure data interpretation. Some existing data on the hatching pattern of resting eggs of crustaceans might further support the predictions of our model. For instance De Stasio [46] reported almost linear increase of the cumulative hatching proportion of diapausing eggs of the freshwater calanoid copepod *Onychodiaptomus sanguineus* (Calanoida: Diaptomidae) over time (months). This planktonic crustacean from astatic water bodies has a relatively short generation time (weeks) thus months of incubation may cover a few generations of their active forms. The linear increase in the cumulative hatching proportion of resting eggs over time may indicate a constant hatching proportion per unit of time (e.g. generations), which agrees with the results of our simulations at considerable environmental variability and low mortality of dormant forms (S5 Fig, S6 Fig). A similar pattern of hatching dynamics of resting eggs with storage time was reported in a freshwater crustacean, *Daphnia magna* (Branchiopoda: Daphnidae) originating from temporary urban ponds [47], as well as in some rotifers [48].

A relatively large data set exists on the hatching phenology of another group of crustaceans–anostracans inhabiting waterbodies of a various range of variability of environmental conditions. At one end of this spectrum are anostracans from permanent (e.g. *Artemia salina—*Branchiopoda: Artemidae [49]) or seasonally occurring waterbodies (e.g. *Eubranchipus grubii—*Branchiopoda: Chirocephalidae [50]) with a near 100% hatch rate at first favourable period. At the other end are species inhabiting unpredictable, ephemeral waters. The hatching fraction of the resting eggs of *Branchipodopsis wolfi* (Branchiopoda: Branchipodidae) originating from Bostwanian episodic rock pools remained at an almost constant level [51], while those of *Branchinecta sandiegonensis* (Branchiopoda: Branchinectidae) or *Streptocephalus woottoni* (Branchiopoda: Streptocephalidae) collected from Californian ephemeral vernal pools declined over subsequent hatching trials [52]. The diversified bet-hedging hatching phenology of the resting eggs may even be observed in vertebrates, e.g. in annual killifish *Nothobranchius furzeri* (Actinopterygii: Nothobranchiidae) inhabiting ephemeral pools in Africa. In this fish, the cohort of resting eggs hatched in decreasing proportions in subsequent hatching trials [53]. We have observed the above spectrum of hatching phenologies in our simulations by merely having manipulated two parameters: the environmental variability and the mortality of dormant forms.

More experimental data is needed to verify other predictions of our model, however.

A diversified duration of developmental arrest is not the only mechanism for coping with temporal fluctuations of environmental conditions. As we have mentioned already, an alternative remedy may be high tolerance of environmental changes, efficient mechanisms of spatial dispersal [38, 54] or in some circumstances also iteroparity [55]. Thus, we may expect the evolution of diversified bet-hedging diapause in fragile short living organisms that have low abilities for dispersal in unpredictably changing habitats primarily. The described phenomenon of the prolonged diapause evolution may likely apply to various kinds of replicators, not only biotic (various organisms including pathogenic microorganisms or rebellious parts of organisms e.g. cancer cells or transposones) but also abiotic (memes, computer viruses, financial investments) ones that compete for limited resources in unpredictably varying habitats.

## Supporting information

S1 Table Experimental variables of the simulations. (DOC)

S1 Fig Effect of various mutation rates on evolution of competing life strategies that differed in lifespan of developmental arrest at moderate fluctuations of the carrying capacity, when K = 500 and SD of K = 500, and mortality of dormant forms = 5% per generation. The thick black line indicate most common value that was used in simulations. Note marginal effect of the mutation rate below 0.00001 on evolution of life strategies.
(DOC)

S2 Fig The probability of drawing particular value of environment capacity in the growing season for different variants of the experiments (for K = 500 and δ–a value of one standard deviation of K defined on each graph). In red are marked years with null chance for reproduction of active forms.
(DOC)

S3 Fig Effect of various carrying capacities on evolution of most successful life strategies, at comparable relative population density fluctuations, i.e. when SD of K = K and mortality of dormant forms = 5% per generation while mutation probability of competing life strategies = 0.00001. The black thick line with triangles indicate most common value used in simulations. Note marginal effect of K on the evolution of life strategies except for very low values of K<25.
(DOC)

S4 Fig Mean survivorship of various life strategies competing for limited resources for 5,000 generations at different range of environmental variability when mortality of dormant forms assumed as 2% per generation. The strategies differ in maximum lifespan of developmental arrest of the diapausing forms. Population fluctuations are presented as relative values of standard deviations of the carrying capacity. For comparison with the Fig 2 in the manuscript where lower survivorship of dormant forms was assumed.
(DOC)

S5 Fig Another example of a final structure of offsprings formed at the end of simulation by the successful life strategies at high population fluctuation SD = 5K and low—1% mortality of dormant forms per generation. Note here, almost equal proportion of offsprings remaining in diapause for different period of time formed by most life strategies. The most successful strategy appeared here the strategy number 18 (indicated by dark grey colour).
(DOC)

S6 Fig Cumulative (added up to 100%) proportion of dormant stages, inactive for various number of generations (0–49) formed by all surviving life strategies at the end of the competition experiments at a moderate range of population fluctuations (SD = K) and various mortalities of dormant forms. (DOC)

S7 Fig Mean survivorship of competing life strategies after 5,000 generations at constant carrying capacity (K = 500, SD = 0), when assumed mortality of dormant forms = 0% per generation. Note that life strategies with ELDs were not outcompeted at these circumstances.
(DOC)

S8 Fig Effect of various fecundities on evolution of life strategies that differed in lifespan of developmental arrest at moderate fluctuations of environmental carrying capacity, when K = 500 and SD of K = K, mortality of dormant forms = 5% per generation, and mutation probabilities of life strategies = 0.00001 per generation. The black thick line with triangles indicate most common value used in simulations. Note marginal effect of fecundity on the evolution of life strategies except very low values of E≤2.
(DOC)

S1 Figure Data Data used to draw all figures in the manuscript. (ZIP)
