# Supplementary material for: How long to rest in unpredictably changing habitats?
Source: PLoS One. 2017 Apr 18;12(4):e0175927. doi: 10.1371/journal.pone.0175927 (PMC5395243; doi:10.1371/journal.pone.0175927)
Supplement: S1 Table — (DOC) [file pone.0175927.s001.doc]

**Supporting Information**

S1 Table. Experimental variables of the simulations

**N_0_** - the initial size of the population in all simulations = 1000 individuals,

**E** - the number of offspring produced in a single season by one individual (as long as it survived until reproduction), in most simulations = 6,

**P_E_** - mortality of dormant offspring over one generation = 5% per generation in most simulations,

**P_M_** - the mutation probability, in all simulations = 0.00001,

**K** - environmental capacity - the mean number of individuals which were able to reproduce in each generation, one of the tested parameters,

**SD or δ** - environmental variability - the relative value indicating standard deviation of normal distribution of variability of environmental capacities, one of the tested parameters.

The last two parameters determine the probability distribution of environmental capacity in the following season.
