# Supplementary material for: How long to rest in unpredictably changing habitats?
Source: PLoS One. 2017 Apr 18;12(4):e0175927. doi: 10.1371/journal.pone.0175927 (PMC5395243; doi:10.1371/journal.pone.0175927)
Supplement: S1 Fig — The thick black line indicate most common value that was used in simulations. Note marginal effect of the mutation rate below 0.00001 on evolution of life strategies. (DOC) [file pone.0175927.s002.doc]

**Supporting Information**

S1 Figure. Effect of various mutation rates on evolution of competing life strategies that differed in lifespan of developmental arrest at moderate fluctuations of the carrying capacity, when K=500 and SD of K=500, and mortality of dormant forms = 5% per generation. The thick black line indicate most common value that was used in simulations. Note marginal effect of the mutation rate below 0.00001 on evolution of life strategies.
