# Supplementary material for: How long to rest in unpredictably changing habitats?
Source: PLoS One. 2017 Apr 18;12(4):e0175927. doi: 10.1371/journal.pone.0175927 (PMC5395243; doi:10.1371/journal.pone.0175927)

**Supporting Information**

S2 Figure. The probability of drawing particular value of environment capacity in the growing season for different variants of the experiments (for K = 500 and δ – a value of one standard deviation defined on each graph). In red are marked years with null chance for reproduction of active forms.


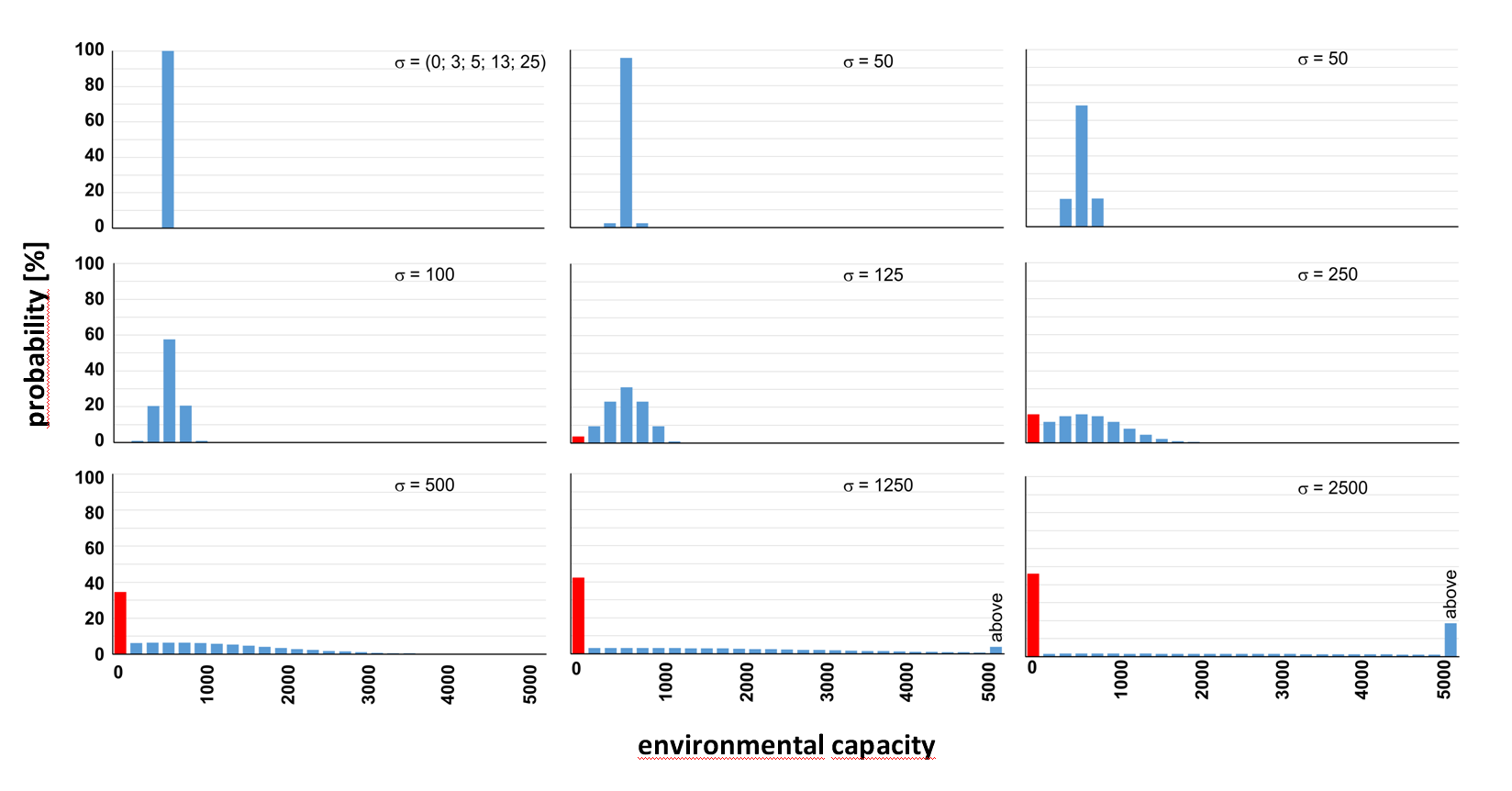

Supplement: S2 Fig — In red are marked years with null chance for reproduction of active forms. (DOC) [file pone.0175927.s003.doc]
