# Supplementary material for: How long to rest in unpredictably changing habitats?
Source: PLoS One. 2017 Apr 18;12(4):e0175927. doi: 10.1371/journal.pone.0175927 (PMC5395243; doi:10.1371/journal.pone.0175927)
Supplement: S3 Fig — The black thick line with triangles indicate most common value used in simulations. Note marginal effect of K on the evolution of life strategies except for very low values of K<25. (DOC) [file pone.0175927.s004.doc]

**Supporting Information**

S3 Figure. Effect of various carrying capacities on evolution of most successful life strategies, at comparable relative population density fluctuations, i.e. when SD of K = K and mortality of dormant forms = 5% per generation while mutation probability of competing life strategies = 0.00001. The black thick line with triangles indicate most common value used in simulations. Note marginal effect of K on the evolution of life strategies except for very low values of K<25.
