# Supplementary material for: How long to rest in unpredictably changing habitats?
Source: PLoS One. 2017 Apr 18;12(4):e0175927. doi: 10.1371/journal.pone.0175927 (PMC5395243; doi:10.1371/journal.pone.0175927)
Supplement: S4 Fig — The strategies differ in maximum lifespan of developmental arrest of the diapausing forms. Population fluctuations are presented as relative values of standard deviations of the carrying capacity. For comparison with the Fig 2 in the manuscript where lower survivorship of dormant forms was assumed. (DOC) [file pone.0175927.s005.doc]

**Supporting Information**

S4 Figure. Mean survivorship of various life strategies competing for limited resources for 5,000 generations at different range of environmental variability when mortality of dormant forms assumed as 2% per generation. The strategies differ in maximum lifespan of developmental arrest of the diapausing forms. Population fluctuations are presented as relative values of standard deviations of the carrying capacity. For comparison with the Fig. 2 in the manuscript where lower survivorship of dormant forms was assumed.


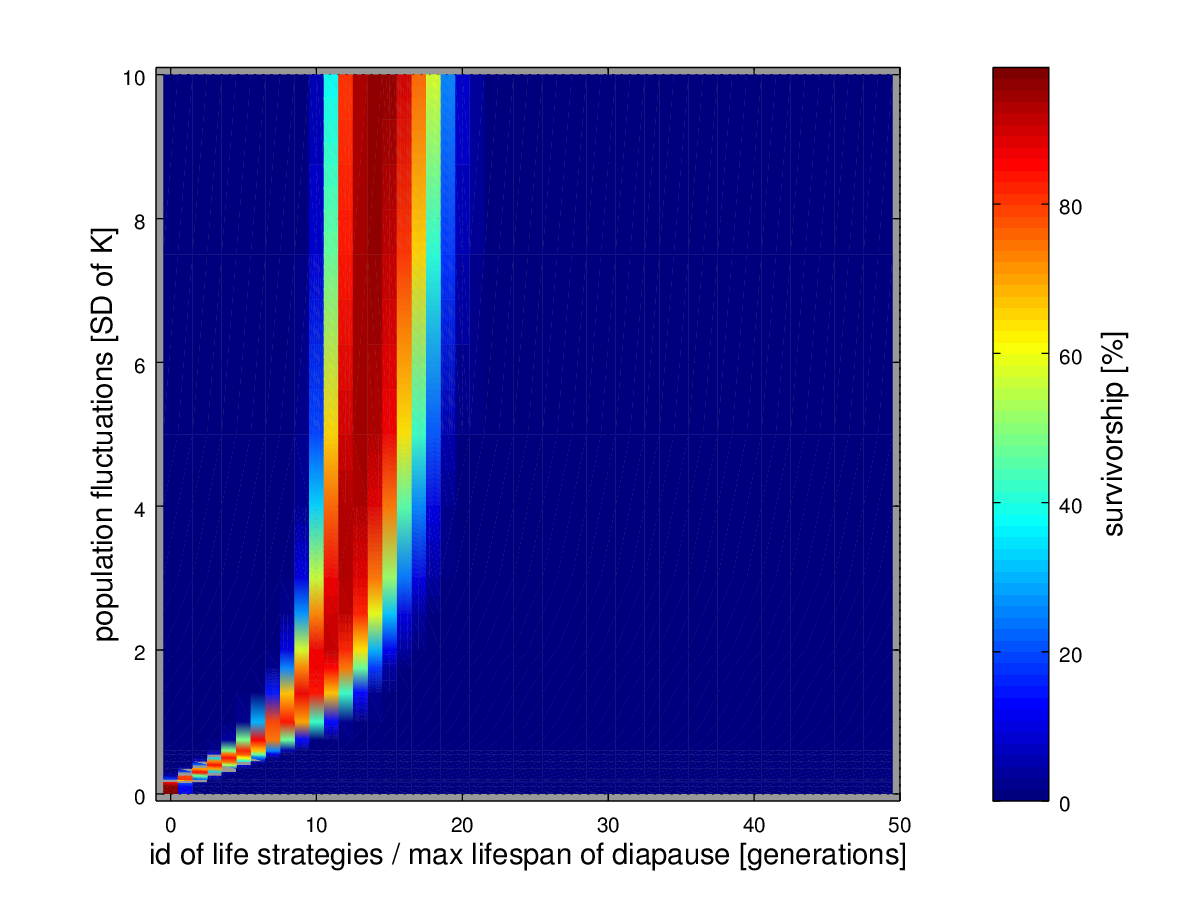


ID of competing life strategies
