# Supplementary material for: How long to rest in unpredictably changing habitats?
Source: PLoS One. 2017 Apr 18;12(4):e0175927. doi: 10.1371/journal.pone.0175927 (PMC5395243; doi:10.1371/journal.pone.0175927)

**Supporting Information**

S5 Figure. Another example of a final structure of offsprings formed at the end of simulation by the successful life strategies at high population fluctuation SD=5K and low - 1% mortality of dormant forms per generation. Note here, almost equal proportion of offsprings remaining in diapause for different period of time formed by most life strategies. The most successful strategy appeared here the strategy number 18 (indicated by dark grey colour).


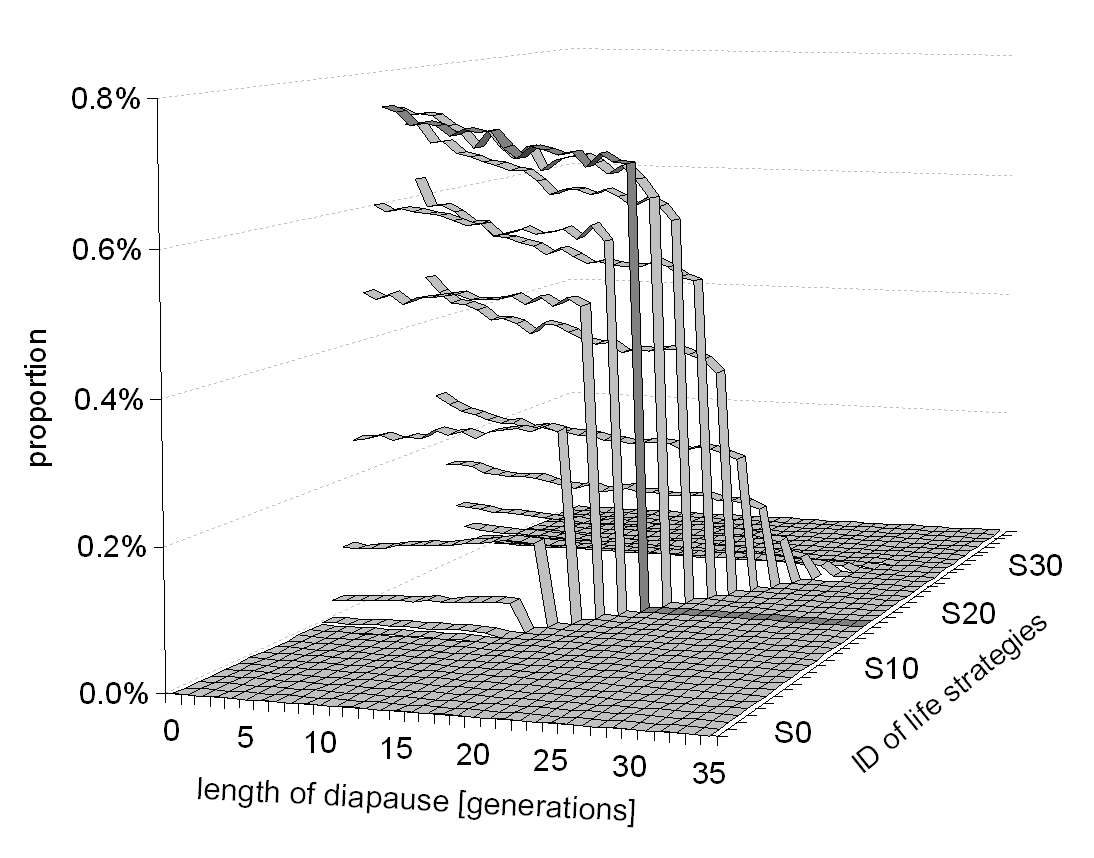

Supplement: S5 Fig — Note here, almost equal proportion of offsprings remaining in diapause for different period of time formed by most life strategies. The most successful strategy appeared here the strategy number 18 (indicated by dark grey colour). (DOC) [file pone.0175927.s006.doc]
