# Supplementary material for: How long to rest in unpredictably changing habitats?
Source: PLoS One. 2017 Apr 18;12(4):e0175927. doi: 10.1371/journal.pone.0175927 (PMC5395243; doi:10.1371/journal.pone.0175927)

**Supporting Information**

S6 Fig. Cumulative (added up to 100%) proportion of dormant stages, inactive for various number of generations (0-49) formed by all surviving life strategies at the end of the competition experiments at a moderate range of population fluctuations (SD = K) and various mortalities of dormant forms.


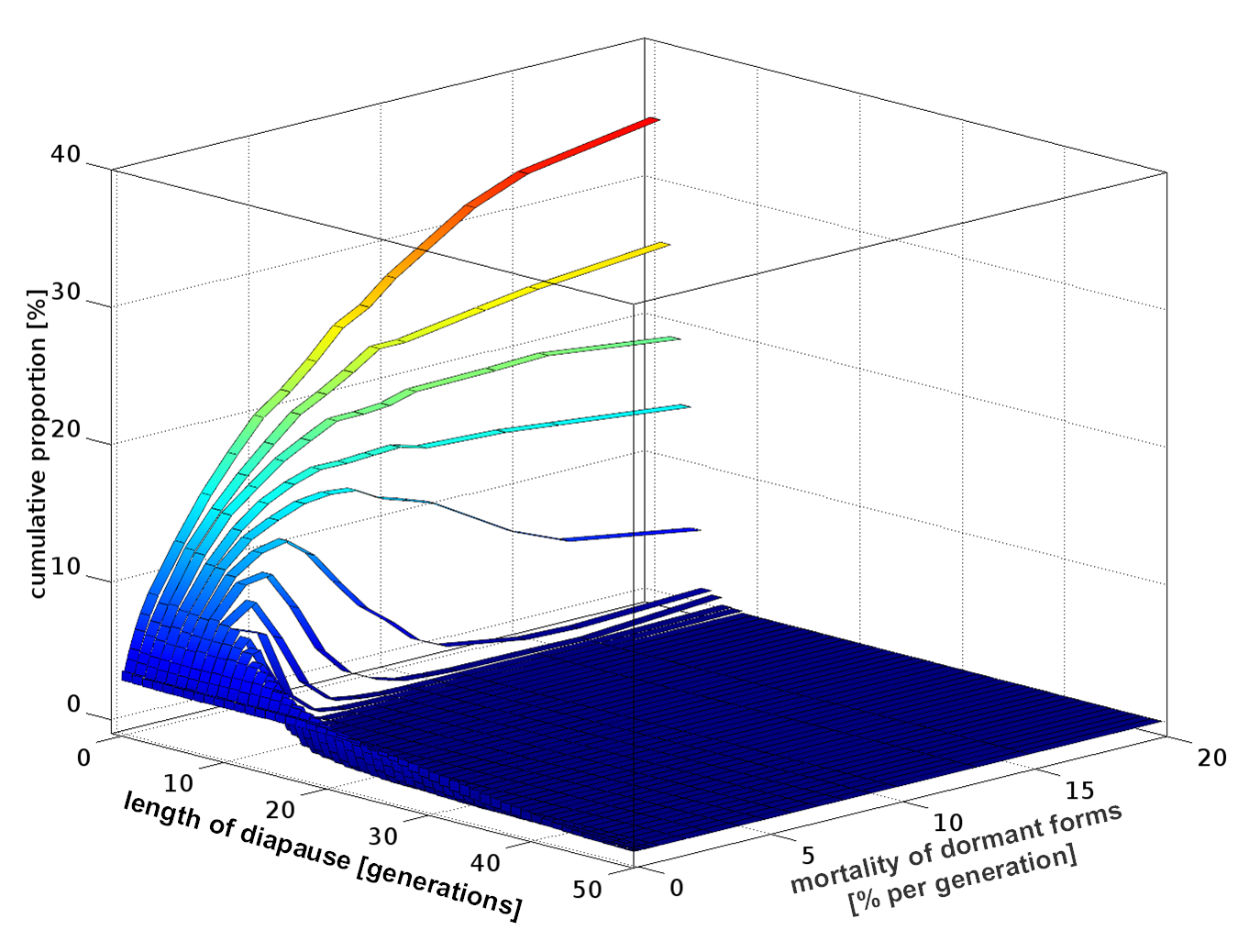

Supplement: S6 Fig — (DOC) [file pone.0175927.s007.doc]
