# Supplementary material for: How long to rest in unpredictably changing habitats?
Source: PLoS One. 2017 Apr 18;12(4):e0175927. doi: 10.1371/journal.pone.0175927 (PMC5395243; doi:10.1371/journal.pone.0175927)
Supplement: S7 Fig — Note that life strategies with ELDs were not outcompeted at these circumstances. (DOC) [file pone.0175927.s008.doc]

**Supporting Information**

S7 Figure. Mean survivorship of competing life strategies after 5,000 generations at constant carrying capacity (K=500, SD=0), when assumed mortality of dormant forms = 0% per generation. Note that life strategies with ELDs were not outcompeted at these circumstances.
