# Supplementary material for: How long to rest in unpredictably changing habitats?
Source: PLoS One. 2017 Apr 18;12(4):e0175927. doi: 10.1371/journal.pone.0175927 (PMC5395243; doi:10.1371/journal.pone.0175927)
Supplement: S8 Fig — The black thick line with triangles indicate most common value used in simulations. Note marginal effect of fecundity on the evolution of life strategies except very low values of E≤2. (DOC) [file pone.0175927.s009.doc]

**Supporting Information**

S8 Figure. Effect of various fecundities on evolution of life strategies that differed in lifespan of developmental arrest at moderate fluctuations of environmental carrying capacity, when K=500 and SD of K=K, mortality of dormant forms = 5% per generation, and mutation probabilities of life strategies = 0.00001 per generation. The black thick line with triangles indicate most common value used in simulations. Note marginal effect of fecundity on the evolution of life strategies except very low values of E≤2.
